# The Trade in African Medicinal Plants in Matonge-Ixelles, Brussels (Belgium)

**DOI:** 10.1007/s12231-016-9365-8

**Published:** 2016-12-16

**Authors:** Tinde van Andel, Marie-Cakupewa C. Fundiko

**Affiliations:** 1 Naturalis Biodiversity Center, Leiden, The Netherlands; 2 Institute of Biology, Leiden University, Leiden, The Netherlands

**Keywords:** African diaspora, Democratic Republic of Congo, Food medicine, Market survey, Medicinal plants, Urban ethnobotany.

## Abstract

Maintaining cultural identity and preference to treat cultural bound ailments with herbal medicine are motivations for migrants to continue using medicinal plants from their home country after moving to Europe and the USA. As it is generally easier to import exotic food than herbal medicine, migrants often shift to using species that double as food and medicine. This paper focuses on the trade in African medicinal plants in a Congolese neighborhood in Brussels (Belgium). What African medicinal plants are sold in Matonge, where do they come from, and to which extent are they food medicines? Does vendor ethnicity influence the diversity of the herbal medicine sold? We hypothesized that most medicinal plants, traders, and clients in Matonge were of Congolese origin, most plants used medicinally were mainly food crops and that culture-bound illnesses played a prominent role in medicinal plant use. We carried out a market survey in 2014 that involved an inventory of medicinal plants in 19 shops and interviews with 10 clients of African descent, voucher collection and data gathering on vernacular names and uses. We encountered 83 medicinal plant species, of which 71% was primarily used for food. The shredded leaves of *Gnetum africanum* Welw., *Manihot esculenta* Crantz, and *Ipomoea batatas* (L.) Lam were among the most frequently sold vegetables with medicinal uses. Cola nuts, shea butter, *Aloe vera* (L.) Burm.f., and *Mondia whitei* (Hook.f.). Skeels were the main non-food medicines sold. Women’s health, aphrodisiacs, and rituals were the most important medicinal applications, but culture-bound ailments did not entirely dominate the plant uses. While most clients in Matonge were Congolese, most vendors and plant species were not. The Pakistanis dominated the food trade, and typical Congolese plants were sometimes replaced by West African species, creating confusion in vernacular names. African-managed shops had significantly more species of medicinal plants in stock than shops managed by Pakistanis. Almost all non-food herbal medicine was sold by Africans. Apart from informal shops, non-food herbal medicine was also sold from private homes and by ambulant vendors, probably to reduce costs and escape taxes and control by the authorities. We expect that in the future, increasing rent, strict regulations, and decreasing investments by the Congolese community will force the medicinal plant trade in Matonge to go even more underground.

**Electronic supplementary material:**

The online version of this article (doi:10.1007/s12231-016-9365-8) contains supplementary material, which is available to authorized users.

## Abstract (French)

**Le commerce des plantes médicinales africaines à Matonge-Ixelles, Bruxelles (Belgique)**. L’Ethnobotanique urbaine, en particulier l’étude des usages des plantes par les migrants en Europe et aux Etats Unis, est une science qui de développe très rapidement. Le souci de maintenir leur identité culturelle et la préférence de traiter des maladies dites de culture par les plantes médicinales, constituent les raisons qui motivent les migrants à continuer à recourir à la pharmacopée traditionnelles de leurs pays d’origine. Comme il est plus facile d’importer les aliments exotiques que les plantes médicinales, les migrants glissent souvent vers les espèces à double usage, à la fois alimentaire et médicinal. Le présent travail se concentre sur le commerce des plantes médicinales Africaines à Matonge-Ixelles, connu comme un quartier populaire Congolais à Bruxelles (Belgique). Quelles plantes médicinales Africaines sont vendues à Matonge, de quelle origine proviennent-elles? Dans quelle mesure ces plantes sont-elles à la fois alimentaires et médicinales? L’ethnicité des vendeurs influence-t-elle la diversité des herbes médicinales vendues? Notre hypothèse était que la plupart des plantes médicinales vendues, les vendeurs et les acheteurs à Matonge étaient d’origine Congolaise, et que les maladies dites de culture joueraient un rôle prépondérant dans l’usage des plantes médicinales vendues. Nous avons conduit des enquêtes sur le marché en 2014 comprenant l’inventaire des plantes médicinales dans 19 points de vente, des entretiens avec 10 clients d’origine Africaine, la collection des specimens, et des données sur les noms vernaculaires des plantes et leurs usages. Nos enquêtes du marché et les entretiens ont permis d’inventorier un total de 83 espèces de plantes médicinales, dont 71% étaient principalement utilisées comme aliments. Les feuilles râpées de *Gnetum africanum* Welw., *Manihot esculenta* Crantz et *Ipomoea batatas* (L.) Lam étaient les légumes les plus vendues comme médicament. Alors que les noix de cola, le beurre de karité, *Aloe vera* (L.) Burm.f. et *Mondia whitei* (Hook.f.) Skeels étaient les principales plantes médicinales non comestibles vendues. La santé des femmes, les aphrodisiaques et les plantes rituelles étaient les plantes médicinales les plus dominantes, alors que les affections dites de culture étaient faiblement représentées dans les usages des plantes. Malgré que la majorité des clients à Matonge soient d’origine Congolaise, la plupart des vendeurs des plantes venaient d’ailleurs. Les Pakistanais dominaient le commerce des aliments et les plantes typiquement Congolaises étaient des fois remplacées par des espèces Ouest Africaines, créant ainsi une confusion des noms vernaculaires. Les magasins gérés par les Africains représentaient plus de plantes médicinales en stock comparativement à ceux gérés par les Pakistanais, et presque toutes les plantes médicinale non comestibles étaient vendues par les Africains. L’inspection des points formels de vente n’a pas couvert l’entièreté de la diversité floristique à Matonge: les plantes médicinales non-comestibles étaient aussi vendues depuis des adresses privées, et par des vendeurs ambulants, probablement pour réduire les couts, les taxes exorbitantes, et le contrôle par les autorités. Nous espérons qu’à l’avenir, l’augmentation du loyer, une réglementation stricte et la diminution des investissements par la communauté Congolaise obligera le commerce des plantes médicinales à Matonge de disparaitre dans l’informel.

## Introduction

Urban ethnobotany, the study of plants used by people in urban environments, in particular among migrant communities, is a rapidly developing field (Ladio and Albuquerque 2016; Vandebroek and Balick 2012). Migrants commonly hold on to their cultural preference of food crops, dishes, and herbal medicines from their homeland, and often maintain traditional concepts on health and illness (Muniz de Medeiros et al. 2012; Pieroni and Vandebroek 2007). This can be seen as a strategy for strengthening their own identity in the host country, but the migrant’s use of medicinal plants can also be motivated by a limited access to modern healthcare due to a lack of insurance or unemployment (Balick et al. 2000; Vandebroek et al. 2007). Culture-bound syndromes (e.g., the ‘evil eye’) are also preferably treated with herbal medicine, as biomedical providers in the host country often do not recognize these ailments (Bayles and Katerndahl 2009; Van Andel and Westers 2010). Migrants often face constraints on the availability of medicinal plant species formerly used in their home countries due to strict laws regarding the import and sale of medicinal plants (Ceuterick et al. 2011; Viladrich 2007; Vandebroek and Balick 2014). As a result, medicinal herbs sold on migrant markets are dominated by food medicines, plants with primarily culinary applications (e.g., fruits, vegetables, staple foods, or spices) that and serve a dual purpose as medicine (Pieroni et al. 2008; Vandebroek and Balick 2012). Moreover, food medicines are mostly widely known species and knowledge about them is more easily exchanged in the multicultural setting of large cities than less familiar, strictly medicinal plants that have a more restricted distribution (Vandebroek and Balick 2014).

Markets are public places, distinctive for each given culture or society, and they represent small-scale reproductions of that region’s cultural and biological diversity (De Albuquerque et al. 2007). As a result, many exotic plant food and medicines can be found on multicultural urban markets. Recently, ethnobotanists have studied the medicinal plant trade and use by migrants in European urban areas, such as Turks in Germany (Pieroni et al. 2005), Thais in Sweden (Lundeberg 2007), Surinamers in the Netherlands (van Andel et al. 2007; van Andel and Westers 2010), Senegalese in Italy (Ellena et al. 2012), and Latinos (Ceuterick et al. 2011), Sikhs (Sandhu and Heinrich 2005) and Pakistanis in the UK (Pieroni et al. 2008). The present study focuses on the medicinal plant trade in the predominantly Congolese neighborhood Matonge-Ixelles in Brussels, Belgium. Originally, the name of a famous center for business, music, and nightlife in Kinshasa, the capital of the Democratic Republic of Congo (former Zaire), the Belgian Matonge represents an international meeting place for African migrants in Brussels. Although Belgium did inherit a trusteeship over Rwanda and Burundi after World War II, Congo was Belgium’s only colony until its independence in 1960. The violent civil war and the general collapse of state control in the Democratic Republic of the Congo (DRC) in the 1990s caused a massive migration of Congolese refugees, of which ca. 70,000 are now estimated to live in Belgium, where they form the largest group of Sub-Saharan immigrants (Demart 2013a; Swyngedouw and Swyngedouw 2009). In the 1970s, after the opening of the Congolese student hostel *La Maison Africaine*, followed by a nightclub, food shops, and galleries around the *Chaussé de Wavre*, this Ixelles neighborhood received the names *Le Petit Congo* and *Le Petit Matonge de Bruxelles*. Nowadays, Matonge is known as a vibrant, multicultural meeting place for migrants, tourists, businesspeople, and young Belgians that come to shop, eat, and party in the numerous African hair and beauty salons, wax print fabric shops, exotic groceries, restaurants, and music bars (Beddington 2013). On the other hand, Matonge has the reputation as being unsafe. After several street riots in 2001 and in the winter of 2011–2012, problems with drug dealers and gang fights, the neighborhood remains under intensified police control (Demart 2008, 2013b). Apart from an overview of non-timber forest products sold on the French and Belgian markets (Tabuna 1999), no studies in urban ethnobotany exist for the African diaspora in Belgium. In this paper, we addressed the following questions: (1) What African medicinal plants are sold in Matonge and for which diseases are they used? (2) To which extent can these plants be characterized as food medicines? (3) Which ethnicities are involved in the trade of African medicinal plants in Matonge? (4) Does vendor ethnicity influence the floristic diversity of the herbal medicine sold?

We assumed the herbal medicines, traders, and clients in Matonge to be predominantly of Congolese origin. We further hypothesized that Congolese migrants in Matonge would mainly buy medicinal plants to treat culture-bound illnesses for which no synthetic treatment is available in Belgium. Finally, because it is easier to import and sell food plants in Europe than herbal medicine, we expected that most medicinal plants sold in Matonge fall within the category food medicines.

## Methods

**Fig. 1:**
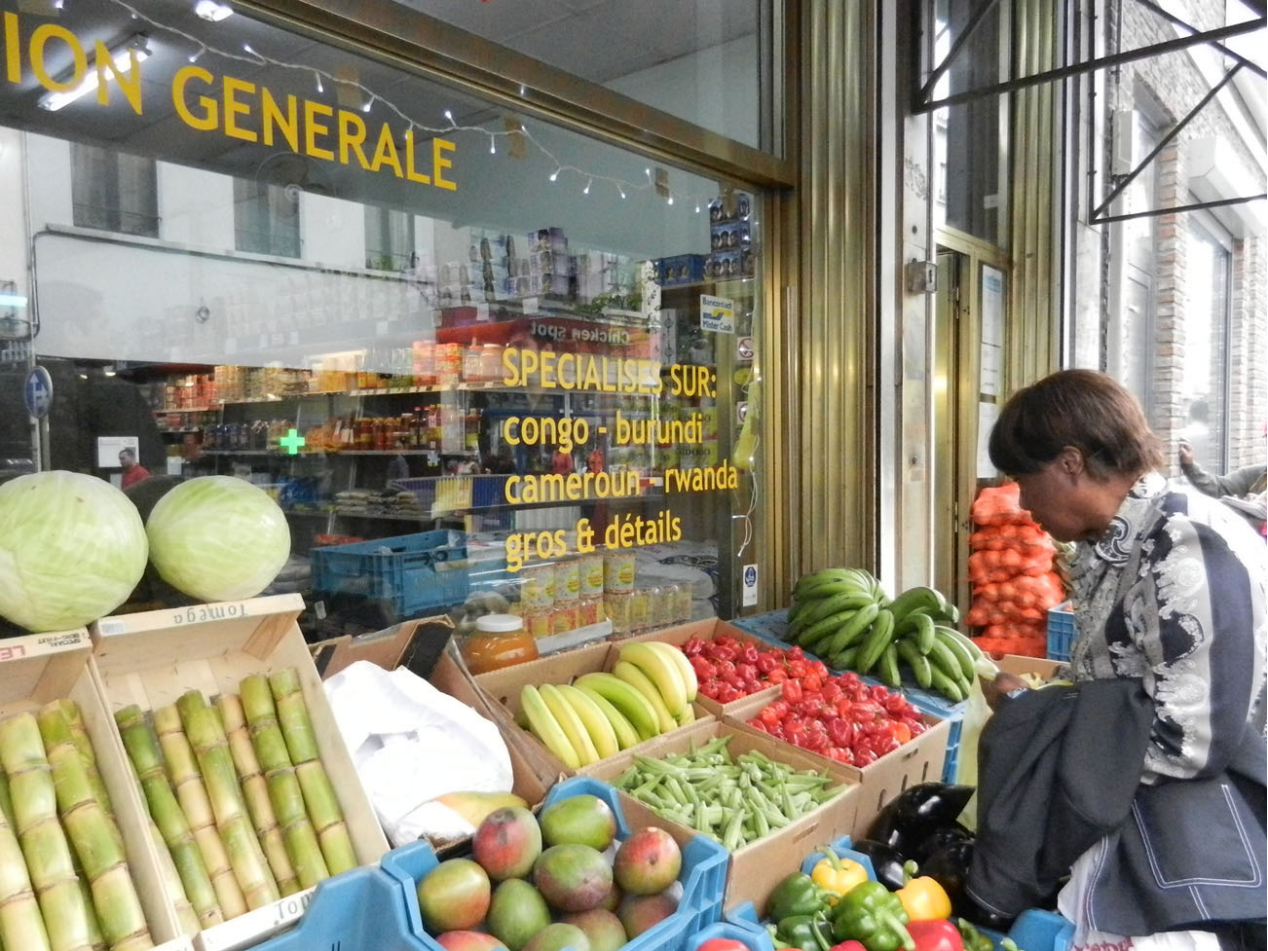
African food shops along the main commercial street at Matonge. Picture by M.C.C. Fundiko.

A market survey was carried out in the neighborhood of Matonge-Ixelles, near the Metro station *Porte de Namur* on the eastern fringe of Brussels city center, on several days between March and June 2014. We counted the number of shops and market stalls that sold African medicinal plants in the area around the *Chaussé de Wavre* (French) or *Waversesteenweg* (Dutch), Matonge’s main commercial street located around 50°49′17″ N, 4°24′24″ E (Fig. 1). We entered shops selling African food and inventoried whether they were selling medicinal plant products and vegetables that doubled as medicine. We counted the number of medicinal plant species per shop. The proportion of food- and non-food medicine was calculated afterwards. We obtained information on vernacular names, preparation methods, and medicinal and food uses from both shopkeepers and clients.

Short semi-structured interviews held with several shopkeepers included questions on their ethnicity, species of medicinal plants sold, type of clients, and provenances of the plant product sold. Questionnaires were held by the second author in English, French, or in the Congolese languages Lingala and Swahili, according to the preferred language of the participant. Additional semi-structured interviews were held among ten African migrants who regularly bought herbal medicine at Matonge. We asked them to name the (vernacular) names of the species they bought, their motivation to buy medicinal plants, how they purchased the herbal medicine (from shops or via the informal circuit), and for which ailments they used these plants. Prior informed consent was obtained from all plant vendors and consumers before the interviews and the collection of data on plant names and uses.

Plant specimens that could not be identified on the spot were purchased, processed into botanical vouchers, identified, and deposited at the herbarium of Naturalis Biodiversity Center (L) at Leiden, the Netherlands. Scientific names were checked with the Plant List (www.theplantlist.org). When vernacular plants names were mentioned during interviews but the particular species was not available in the shops, or when specimens lacked sufficient morphological features for identification, we verified their vernacular names with literature (e.g., Daeleman and Pauwels 1983; Schmelzer and Gurib-Fakim 2008, 2013; Tabuna, 1999; Termote et al. 2012) and online databases, such as the PROTA Database (www.prota4u.org) and the Prelude Medicinal plant database (www.africamuseum.be/collections/external/prelude). To compare the floristic diversity of medicinal plants in shops managed by Africans with shops managed by non-Africans, we first used a Kolmogorov-Smirnov test to see whether our data were normally distributed. When this was the case, we compared the mean number of plant species sold by African and non-Africans by means of a one-sample *t* test. All statistical tests were done in the program SPSS 19.

## Results

### Traded Species

In the 19 shops in Matonge that sold African plants, we recorded ca. 69 species and collected 54 specimens, of which one wood/bark specimen remained unidentified and six species could only be identified to genus level (Electronic Supplementary Material (ESM), Appendix 1). We conducted ten interviews with African clients that regularly bought herbal medicine in Matonge (four males, six females, 20–65 years old, migrants from the DRC, Burundi, Burkina Faso, Senegal, Mauritania, and Cameroon, living in Belgium, France, and the Netherlands). During those interviews, an additional 14 plant species were mentioned to have been bought in Brussels, but were not available in the shops at the time of our fieldwork; two of which were of sufficient quality to make botanical vouchers. All plant species are listed with their family, scientific and vernacular name, voucher numbers, plant parts, and food and medicinal uses in ESM.

The most species-rich families were the Leguminosae (nine spp.), followed by the Malvaceae (eight spp.), Apocynaceae (five spp.), and Solanaceae (five spp., *Solanum aethiopicum* L. with two different cultivars). The Leguminosae is a very large family and thus contains many medicinal species, but the less diverse families of Apocynaceae, Solanaceae, and Malvaceae contain a disproportionally high percentage of species with therapeutic value (Moerman 1991). In total, ca. 83 plant species were either encountered in the shops or were said to have been bought there, of which 57 (71%) were used both for food and medicine. Table 1 lists the 15 most frequently sold plant species in Matonge, calculated as the percentage of shops in which we encountered the species. Apart from the seeds of *Cola nitida* (Vent.), Schott and Endl. (cola nut) and *Garcinia kola* Heckel (bitter cola nut), which are chewed as stimulants and aphrodisiacs, all these frequently sold species are used primarily as food.

**Table 1:** The top 15 of most frequently sold plant species used as food medicine in 19 shops in Matonge, Brussels (March–June 2014).

Species	Plant part	Percentage
*Gnetum africanum*	Sliced leaves (frozen or dried)	89%
*Elaeis guineensis*	Oil from fruits	84%
*Colocasia esculenta*	Starchy tuber	84%
*Capsicum annuum*	Fresh and dried fruits	79%
*Manihot esculenta*	Starchy tuber and frozen leaves	79%
*Abelmoschus esculentus*	Fresh fruits	79%
*Ipomoea batatas*	Fresh leaves	74%
*Musa* × *paradisiaca*	Fresh fruits	68%
*Hibiscus acetosella*	Fresh and dried leaves	32%
*Hibiscus sabdariffa*	Fresh leaves, dried calyces	26%
*Aloe vera*	Fresh leaves	26%
*Zingiber officinale*	Fresh rhizomes	26%
*Dacryodes edulis*	Fresh fruits	21%
Cola nitida*	Fresh seeds	21%
Garcinia kola*	Fresh seeds	21%

*Species not primarily sold as food

**Fig. 2:**
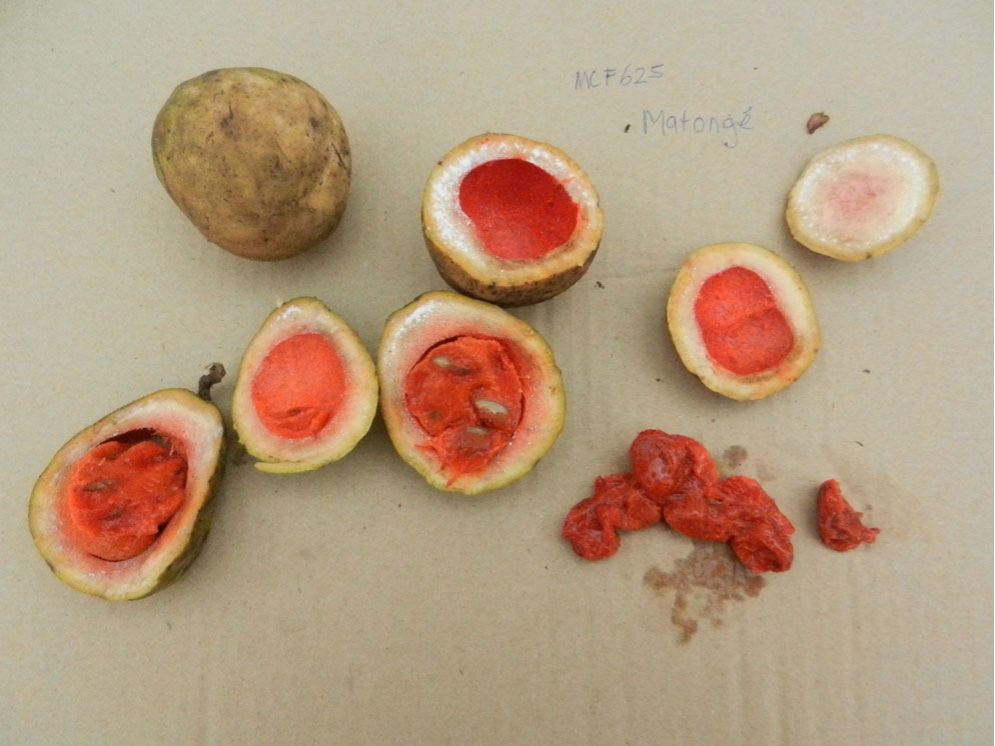
Fresh Matonge fruits (*Landolphia owariensis*), a popular, wild-harvested fruit in the DRC. Picture by M.C.C. Fundiko.

Typically, the fresh, sour fruits of the rainforest liana *Landolphia owariensis* P. Beauv. (Apocynaceae) were also offered for sale (Fig. 2). They are known among the Congolese as ‘Matonge’ in Lingala, and it was after these fruits that the Kinshasa nightlife quarter (and eventually the Brussels neighborhood) was originally named. The fruits are said to be very healthy and should be eaten by people suffering from bronchitis.

We observed that the somewhat similar-looking and tasting fruits of the West African savanna liana *Saba senegalensis* (A. DC.). Pichon were also marketed as ‘Matonge’ in two shops, although this species does not occur in Congo and was probably imported from Mali. Some Congolese, however, recognized the *Saba* fruits as different from *Landolphia* and called them ‘Makalakonki’ (Kikongo), a name used in the DRC for the edible fruits of *Strychnos* spp. that have a similar color and size.

### Medicinal Applications

**Fig. 3:**
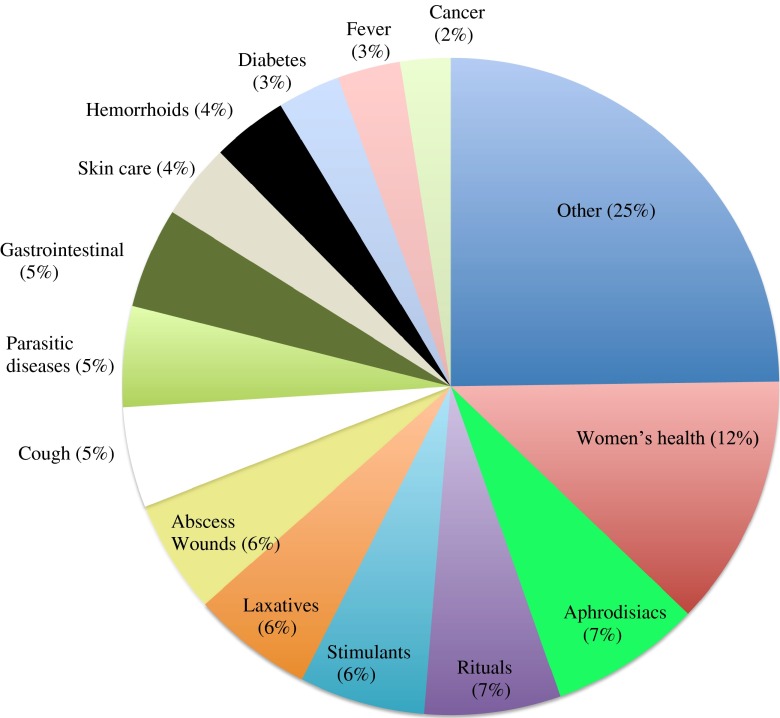
Most frequently mentioned disease categories, expressed as percentage of the total number of plant species.

Clients and shopkeepers mentioned a total of 162 medicinal uses for a wide variety of ailments, of which the most frequent are visualized in Fig. 3. Women’s health was a major category: plants were used to ensure a healthy pregnancy, ease birth, treat postpartum hemorrhage, and induce abortion. Twelve species were mentioned as aphrodisiacs for men, several of which were also used as general stimulants. Although diabetes seemed a minor category with only five plant species listed for treatment, two of these species were among the top three of the most frequently sold food medicines. The same accounts for hypertension, treated by the lemonade made from *Hibiscus sabdariffa* L., of which the calyces were sold in more than 25% of the shops and by onions, commonly available in supermarkets throughout the country (ESM).

Rituals also played a role in the trade in African plants: the fragrant bark of *Commiphora* sp. and the perfumed rhizomes of *Cyperus esculentus* L. were burnt inside the house to chase away bad spirits. The fresh leaves of *Ocimum gratissimum* L. served as essential ingredients in the ritual dish *Tschilwabenye*, cooked with chicken and palm oil and served to the in-laws of the young bride during marriage ceremonies of the Congolese Baluba ethnic group. One client lamented the recent death of a Congolese traditional healer, active in Matonge and Clemenceau, a nearby Brussels neighborhood, who was a specialist in the *Beta Zeke* ritual, which includes throwing shells to predict people’s future and their luck in travel, health, and employment.

### Provenance of Plants

The interviews with vendors and clients revealed that the origin of the plants sold at Matonge was more diverse that previously anticipated. Each vendor had his or her own providers and network. Apart from the DRC, the most cited countries of origin of the plant material were Cameroon, Ghana, Senegal, Benin, Mali, South Africa, and Uganda. Some herbal medicine and food plants bought by African customers were also imported from Brazil, Colombia, or Thailand. When we compared our species list with the literature on medicinal plants used in the DRC, we found nine species that did not occur in the DRC, but were typical medicinal plants of the West African dry zones (Arbonnier 2004): *Acacia nilotica* (L.) Delile, *Annickia polycarpa* (DC.) Setten and Maas ex I.M.Turner, *Commiphora* sp., *Cyperus esculentus*, *Saba senegalensis* and *Ziziphus mucronata* Wildd., *Combretum micranthum* G.Don, and *Combretum* sp. The latter two species were marketed as the medicinal tea *Kenkeliba*. All these species carried Pulaar and/or Wolof names, and were probably imported from Senegal or Mali, just like the unidentified bark sample MCCF653, known as *Dadi* (Pulaar) and boiled as tea against stomachache. The clients that we interviewed did not buy all their medicinal plants at the African shops in Matonge. They also ordered plants directly by air parcels from their family and friends in Africa, from Congolese migrants in Brussels who sold their herbal medicine from their private garages or even delivered at the client’s home, and from ambulant vendors who were said to walk into restaurants, barbershops, and African beauty salons in Matonge with bags of medicinal plants. According to our respondents, these ambulant vendors sold specific types of African herbal medicine (e.g., strong aphrodisiacs) that were difficult to find in the official food shops. This informal trade system probably explains the discrepancy in species mentioned during interviews and observed in shops.

### Vendor Ethnicity and Diversity in Herbal Medicine

Just six of the 19 shops selling African food medicine were managed by African migrants (one Mauritanian, one Senegalese, two Cameroonians, and three Congolese). The remaining 13 shops (68%) were all managed by Pakistanis. After proving that our data were normally distributed, the one-sampled *t* test showed that the shops managed by Africans had a significant higher number of medicinal plant species in stock (an average of ca. 20 species) than those managed by Pakistanis, who sold an average of ca. 11 species (*t* = 6.623, *p* = 0.000). Besides the two species *Garcinia kola* and *Cola acuminata*, 24 of the 26 (92%) of non-food herbal medicines were encountered in African-managed sale points.

In general, the Pakistani shopkeepers were very reluctant to answering questions and often said they did not know any medicinal applications for their plant products. The African vendors, however, were also uncomfortable in providing plant use information. A Mauritanian shopkeeper, who sold a variety of West African medicines, explained his hesitance as follows: “There are difficulties that we encounter during the provision and the trade in plant products.” A Congolese vendor said she “could not give any interview due to security problems.” Another shopkeeper from the DRC, who had lived in Matonge for 40 years, said that Congolese migrants were nowadays less interested in the trade in food or medicine due to high taxes, unaffordable rent and “the regulations.” A Congolese customer argued that the tense police control in the neighborhood and the negative impact of the migrants’ activities had led to the closing of several Matonge selling points by the authorities.

### Motivations to Buy African Traditional Medicine at Matonge

Regardless of the problems faced by the vendors while running their businesses and the apparent need to keep their commercial network secret from the authorities, Congolese migrants, also those living in France or the Netherlands, still constitute the most important group of customers at Matonge. The shops managed by Africans also served as multicultural meeting places, where knowledge on traditions and herbal medicine was still being shared among vendors and clients. Customers said they bought food and medicine at Matonge to strengthen their cultural identity and wanted to encourage African vendors to spread their traditions and culture in Europe. Several clients said that traditional medicines were more effective than modern medicine, such as ground pumpkin seeds (*Cucurbita pepo* L., of the *Mbika* cultivar), consumed to treat prostate cancer. Some illnesses were said to be “typically African” and “ignored by European physicians.” An example was the Congolese cultural bound illness *Mpese*, a severe type of eczema and skin infection that was “caused by enemies who throw the sickness on someone” and “usually very difficult to be treated by synthetic medicine.” Finally, a Congolese woman living in the Netherlands said she preferred medicinal plants above pills, since “the herbal medicine from my country does not contain harmful elements such as pesticides, because these plants are collected in the forest.”

## Discussion and Conclusions

Matonge offers a variety of medicinal plant species to its African customers, but the sale in herbal medicine remains somewhat hidden. While food medicines are openly offered for sale, non-food medicines are sold almost exclusively by African-managed shops, which keep them under the counter or conceal them wrapped in newspaper, by ambulant vendors and illegal shops in private homes or garage boxes. For several reasons, our market survey has only uncovered part of the entire diversity of African traditional medicine sold in this multicultural neighborhood. Powdered and fragmented medicinal plant products, herbal extracts, dried caterpillars, and edible fungi sold at Matonge were excluded because of identification problems. Moreover, due to the informal (and largely invisible) trade and the general reluctance of providing medicinal uses for plants, we must have missed several species of herbal medicine sold “under the counter.”

Culture-bound illnesses certainly played a role in the demand for herbal medicine in Matonge. The popularity of aphrodisiac plants is also known from West African (Quiroz et al. 2014; Van Andel et al. 2012) and Central African markets (Biloso and Lejoly 2006; Termote et al. 2012a; Towns et al. 2014) and the Afro-Caribbean (Van Andel et al. 2012b), where these bitter tonics are regarded as a part of one’s manliness and cultural identity rather than just as treatments for impotence.

The category “women’s health” also represented several cultural bound health beliefs often recorded among Africans and their diaspora, such as the use of herbs for vaginal baths (Van Andel et al. 2008) and specific food to ensure a healthy pregnancy (Towns and Van Andel 2016). Some of the plants marketed at Matonge could cause serious health risks, like the highly poisonous *Erythrophleum africanum* (Benth.) Harms (Kawanga 2006). The powdered bark of this tree is macerated with red earth and palm oil (*Elaeis guineensis* Jacq.) and rubbed on the body of nursing mothers who have just delivered in order to rejuvenate their skin.

Chronic diseases like diabetes and hypertension are increasingly prevalent among Sub-Saharan Africans and their diaspora, and caused by a change in lifestyle (different diet and less physical exercise) after migration from rural to urban areas (Beune et al. 2006; Mbanya et al. 1998, 2010; Vandebroek and Balick 2014). These illnesses were not that visible in our market survey with regard to the number of species used and the specific applications mentioned by our informants. We suggest, however, that future studies should focus on the potential of popular bitter African vegetables (e.g., *Gnetum africanum* Welw., *Solanum* spp., *Momordia charantia* L., and *Vernonia amygdalina* Delile) for diabetes prevention among migrants, as some of these have proven anti-diabetic effects (Atangwho et al. 2014; Van Andel and Carvalheiro 2013). The same accounts for vegetables consumed to lower hypertension, such as the two species mentioned by our informants (onions and *Hibiscus sabdariffa*), which both show activity against high blood pressure (Gbolade 2012; Walton et al. 2016). Although not relevant for the Belgian health situation, plant uses to combat tropical diseases such as malaria and intestinal parasites were still mentioned during our interviews, probably because they remain relevant for migrants frequently visiting their homeland.

While most of the customers in Matonge are migrants from the DRC, the traders and the herbal medicine itself are not necessarily Congolese. Traders can therefore not always meet the demand for specific Central African plants, which results in the substitution of highly valued Congolese plants by similar-looking West African species and a subsequent mix-up in local names, as was illustrated by the replacement of the original Matonge fruit (*Landolphia owariensis*) by *Saba senegalensis*. Substitution of useful plants from the home country by “new” species in the host country is a common aspect of migrant ethnobotany (Muniz de Medeiros et al. 2012), as is the associated confusing mixture of retention, change and replacement of local names (Otieno et al. 2015; Van Andel et al. 2014).

When we compared our results with studies on Congolese medicinal plant use (Biloso and Lejoly 2006; Fundiko 1997; Katemo et al. 2012; Makumbelo et al. 2008; Mpiana et al. 2007; Tabuna 1999; Termote et al. 2012; www.prota4u), we found that 80% of the species we encountered in Matonge are also used as medicinal plants in Congo, while the remaining 20% are typical West African species. However, several of the most commonly sold non-food medicinal plants in Kisangani (Katemo et al. 2012) and Kinshasa (Makumbelo et al. 2008), two major cities in the DRC, were not found in Matonge, such as *Alchornea cordifolia* (Schumach. and Thonn.) Müll.Arg., *Annona senegalensis* Pers., *Bridelia ferruginea Benth.*, *Hymenocardia acida* Tul., and *Newbouldia laevis* (P.Beauv.) Seem. These species probably find their way to Belgium through the informal circuits.

The dominance of food medicine over non-food medicinal plants in Matonge is not simply explained by the fact that food plants are easier to sell and more widely known than herbal medicine or that it responds to a specific demand of the customers (Ceuterick et al. 2011; Vandebroek and Balick 2012, 2014; Viladrich 2007). The percentage of food-medicine species in the African neighborhood Matonge (71%) was higher than the percentages reported among Dominicans in New York (>50%) (Vandebroek and Balick 2014) and among Colombians (70%), Peruvians (62%), and Bolivians (57%) in London (Ceuterick et al. 2011). The high percentage of food medicines in the African groceries in Brussels also appears a result of regulations inhibiting the marketing of medicinal plants in Belgium and the gradual take over by groceries managed by non-Africans. Although some of these Pakistani shop owners were actually born in the DRC (Swyngedouw and Swyngedouw 2009), their knowledge of (or motivation to engage in) non-food African herbal medicine seems limited. They apparently have better access to international trade networks, investment funds, official permits, and qualifications and/or are more willing to engage in obtaining these than native Congolese (Demart 2013b; Hubo 2015; Vandecandelaere 2012). Moreover, the Pakistani traders probably profited from the closure of several Congolese shops after the riots in 2001 and 2012 (Demart 2008; 2013b).

Shortly after the 1970s, African migrants could no longer afford to live in Matonge (Beddington 2013). With the diplomatic quarter of Brussels nearby, it is likely that rents will only go up in the future, and the now dilapidated apartments in Matonge become attractive for real estate investors to renovate and sell to affluent citizens (Vandecandelaere 2012). Therefore, we expect that although the commercialization in African food medicine will probably go on for some time (or move to a peripheral and cheaper neighborhood), the trade in non-food medicinal plants will go entirely underground. African migrants will continue to sell traditional medicine imported from their home country, but increasingly via the internet, out of view from the general public, taxes, quality and safety control entities, and researchers. The diversity of African herbal medicine and the exchange of ethnobotanical information, now provided by the African-managed shops, will become less accessible to migrants. It remains to be seen for how long Matonge will continue to be a “meeting at home place” for African migrants.

## Electronic supplementary material

Below is the link to the electronic supplementary material.

ESM 1 (XLSX 23 kb)
